# Active MMP-8 Quantitative Test as an Adjunctive Tool for Early Diagnosis of Periodontitis

**DOI:** 10.3390/diagnostics11081503

**Published:** 2021-08-20

**Authors:** Marcela Hernández, Mauricio Baeza, Ismo T. Räisänen, Johanna Contreras, Taina Tervahartiala, Alejandra Chaparro, Timo Sorsa, Patricia Hernández-Ríos

**Affiliations:** 1 Laboratory of Periodontal Biology, Department of Oral Pathology and Medicine, Faculty of Dentistry, University of Chile, Santiago 8380544, Chile; mhernandezrios@odontologia.uchile.cl; 2 Department of Conservative Dentistry, Faculty of Dentistry, University of Chile, Santiago 8380544, Chile; mbaeza.paredes@odontologia.uchile.cl (M.B.); jcontreras@odontologia.uchile.cl (J.C.); 3 School of Public Health, Faculty of Medicine, University of Chile, Santiago 7510040, Chile; 4 Department of Oral and Maxillofacial Diseases, University of Helsinki and Helsinki University Hospital, 00290 Helsinki, Finland; ismo.raisanen@helsinki.fi (I.T.R.); taina.tervahartiala@helsinki.fi (T.T.); timo.sorsa@helsinki.fi (T.S.); 5 Department of Periodontology, Centro de Investigación e Innovación Biomédica (CIIB), Faculty of Dentistry, Universidad de Los Andes, Santiago 7620001, Chile; chaparro.ale@gmail.com; 6 Division of Periodontology, Department of Dental Medicine, Karolinska Institutet, 14152 Huddinge, Sweden

**Keywords:** periodontitis, periodontal disease, matrix metalloproteinase-8, gingival crevicular fluid, biomarkers, diagnosis

## Abstract

Periodontitis is a host-mediated bacterial disease that affects the tooth attachment apparatus. Metalloproteinase-8 (MMP-8), a validated biomarker, could aid in clinical diagnosis. This study aimed to evaluate the diagnostic performance of active (a) MMP-8 immunotest versus total (t) MMP-8 ELISA for quantitative real-time diagnosis and assessment of periodontitis severity at the site level. Gingival crevicular fluid (GCF) was sampled from 30 healthy, 42 mild, and 59 severe periodontitis sites from thirty-one volunteers. MMP-8 concentrations were determined by time-resolved immunofluorometric assay (IFMA) and enzyme-linked immunosorbent assay (ELISA). Statistical analysis was performed using the STATA package. Both active and total MMP-8-based methods discriminated among sites according to periodontal diagnosis and severity, with a positive correlation between the two tests (*p* < 0.001). (a) MMP-8 models showed the best performance in receiver operating characteristic (ROC) curves to discriminate between healthy and periodontitis sites (area under the curve [AUC] = 0.89), while (t) MMP-8 demonstrated a high diagnostic precision in the detection of mild from severe periodontitis sites (AUC ≥ 0.80). The use of (a) MMP-8 and (t) MMP-8 could represent a useful adjunctive tool for periodontitis diagnosis and severity. These results support the applicability of new point-of-care methods in the monitoring of high-risk periodontal patients.

## 1. Introduction

Periodontitis is a globally prevalent public health problem that may lead to tooth loss, esthetic and functional impairment, an elevated economic burden, and even higher risk of other noncommunicable diseases, such as diabetes and atherosclerotic events [1]. Furthermore, periodontitis affects the tooth attachment apparatus. It enables the destruction of periodontal ligament fibers, alveolar bone, and apical migration of the gingival junctional epithelium, caused by a dysbiotic microflora that triggers complex immunoinflammatory responses [2].

Periodontal diagnosis is based on traditional clinical measurements of clinical attachment loss, gingival probing depth, and radiographic findings that mainly represent past events of tissue destruction, with a low sensitivity to detect periodontitis at early stages. So, ideal diagnostic methods to screen susceptible individuals and sites, predict future destruction, and monitor periodontal therapy response are still being sought [3]. Molecular biomarkers may aid in the incipient diagnostic accuracy of periodontitis, and the potential incorporation of valid biomarkers was recently proposed in the last periodontal classification system [2].

Oral fluids constitute particularly promising sources to detect molecular markers. Gingival crevicular fluid (GCF) is a serum transudate that leaks through the gingival sulcus, which can be easily and non-invasively obtained, and represent both local and systemic responses [3]. A wide range of periodontal biomarkers have been studied in periodontitis, including inflammatory components and host response modifiers, tissue-breakdown products, and host-derived enzymes [4,5].

Matrix metalloproteinases (MMPs) are a large family of proteases that act in pathological and physiological conditions and together can degrade almost all the components of the extracellular matrix [6,7]. Matrix metalloproteinase-8 (MMP-8) is the leading collagenase in gingival connective tissues, accounting for more than 90% of GCF collagenolytic activity [6,8,9,10]. Its presence in GCF and other oral fluids was associated with periodontal diagnosis, classification, response to treatment, and disease severity [6,7,11,12,13]; thereby, many studies validated MMP-8 as the most effective biomarker in GCF for periodontitis and a spectrum of systemic diseases [14].

MMP-8 activity in periodontal tissues is regulated by a complex interplay between their activators (other MMPs, proteases, and reactive oxygen species) and inhibitors (tissue inhibitors of metalloproteinases) [15]. According to the antibody used and the enzymatic form to target MMP-8, different qualitative and quantitative, laboratory, and point-of-care methods have different levels of precision and agreement as a periodontal biomarker. Whereas enzyme-linked immunosorbent assay (ELISA) and other immunodetection methods identify both latent and active forms of MMP-8, time-resolved immunofluorometric assay (IFMA) detects neutrophil and fibroblast MMP-8 isotypes, mainly in their active forms [16,17,18].

Up to now, the potential role of different tests in the detection of the varying severities of periodontal disease needs to be supported and re-addressed in the new classification framework. This study aimed to evaluate the applicability of active matrix metalloproteinase (a) MMP-8 immunotest versus total (t) MMP-8 ELISA for the quantitative real-time diagnosis and assessment of site severity of periodontitis. We propose that both active and total MMP-8 show potential for periodontal site screening; however, active MMP-8, determined by IFMA, is more accurate for periodontitis diagnosis.

## 2. Materials and Methods

### 2.1. Study Sample and Measurements

#### Cross-Sectional Clinical Study

Eighteen patients with periodontitis and 14 healthy controls were selected from Centers of Diagnostics and Treatment of Northern Metropolitan Health Services, Santiago, Chile. One participant was dropped from the healthy group, and therefore 13 individuals were included and analyzed.

Individuals affected by periodontitis were included if they had at least 14 natural teeth, with a minimum of three remaining molars (excluding 3rd molars); as 5 or more sites with periodontal probing depth (PPD) > 5 mm [19], clinical attachment loss (CAL) > 3 mm and radiographic bone loss [20]. Healthy individuals were selected if they presented bleeding on probing (BOP) < 10% and PPD ≤ 3 mm in every site of the mouth [21]. The following conditions were excluded from the study sample: previous periodontal treatment; systemic disorders such as diabetes mellitus and osteoporosis; pregnancy or nursing; or intake of medications that could affect periodontal tissues within the past three months before the study.

All subjects gave their informed consent for inclusion before they participated in the study. The study was conducted over 4 years, following the Declaration of Helsinki, and the protocol was approved by the Ethics Committee of the Faculty of Dentistry, University of Chile, and endorsed by the FONDECYT (National Fund for Scientific and Technological Development) Bioethics Advisory Committee (FONDECYT 1090046, 28 April 2009).

Demographic variables (age, sex), smoking status, and periodontal clinical measurements were registered in a specially designed chart, by three calibrated examiners (JC, MB, and PH). Periodontal probing depth, clinical attachment loss, and bleeding on probing were recorded in six sites per tooth (mesiobuccal, buccal, distobuccal, distolingual, lingual, and mesiolingual) using a North Carolina probe (UNC-15, Hu-Friedy, Chicago, IL, USA). PPD was defined as the distance, in millimeters, from the gingival margin to the base of the gingivo-dental sulcus, and CAL was defined as the distance from the amelo-cemental junction to the base of the gingivo-dental sulcus. BOP was recorded as present or absent after 15 s. According to the aforementioned method, periodontitis sites (Ps) were classified as follows: mild sites (M, initial to moderate periodontitis), with CAL ≤ 4 mm and PPD ≤ 5 mm, are present in periodontitis stages I and II; (2) severe sites (S, severe to advanced periodontitis), with CAL ≥ 5 mm, are present in stages III and IV [2]. Healthy sites (H), with PPD ≤ 3 mm without BOP, were sampled from healthy individuals [21].

### 2.2. GCF Sampling

GCF samples were collected from M, S, and H sites placing paper strips (Periopaper^®^, ProFlow, Amityville, NY, USA) into the periodontal sulcus until mild resistance was sensed, during 30 s [22]. Strips contaminated by blood or saliva were removed. Two to six GCF samples were obtained from each patient, with 132 sites finally sampled from the 31 volunteers.

As previously reported [20], gingival crevicular fluid was eluted from the strips in a constant ratio of 80 μL of buffer containing 50 mM Tris-HCl pH 7.5, 0.2 M NaCl, 5 mM CaCl_2_, and 0.01% Triton X-100 (Sigma-Aldrich, St. Louis, MO, USA). Eluted samples were frozen at −80 °C until molecular analyses.

### 2.3. MMP-8 Assays

#### 2.3.1. Immunofluorometric Assay

Active MMP-8 concentrations were determined in GCF samples by IFMA, as described by Hemmilä [23]. Briefly, IFMA is based on anti-MMP-8 recognition by the monoclonal MMP-8 specific antibodies 8708 and 8706 (Oy Medix Biochemica Ab, Espoo, Finland), as a catching antibody and a tracer antibody, respectively. The tracing antibody was labeled with europium chelate. The samples were diluted in assay buffer, and after adding an enhancement solution, fluorescence was measured using an EnVision 2105 Multimode Plate Reader (PerkinElmer, Turku, Finland). The specificity of the monoclonal antibodies against MMP-8 corresponded to that of polyclonal MMP-8. The detection limit for the assay is 0.08 ng/mL.

#### 2.3.2. Enzyme-Linked Immunosorbent Assay

Total MMP-8 in GCF was measured by a commercial ELISA assay (Quantikine^®^, R&D Systems^®^ a bio-techne^®^ brand, Minneapolis, MN, USA), following the recommendations of the manufacturer. Briefly, the MMP-8 ELISA assay measures human total MMP-8 (pro- and active MMP-8). Diluted samples were loaded into pre-coated wells of microplate and incubated with total MMP-8 conjugate. The reaction was visualized with substrate solution and the colorimetric reaction was read at a spectrophotometer at 450 nm, using the Victor^TM^ X4 (Wallac Oy, Turku, Finland by PerkinElmer Singapore), with a detection limit of 0.013 ng/mL. Final MMP-8 concentrations were obtained from a standard curve.

### 2.4. Statistical Analyses

The study sample was estimated in a previous study [11,12], with a minimum of 14 individuals per group, considering alpha = 0.05 and 95% power. Categorical variables were presented as frequencies and analyzed using the chi-square test. The normality of the distribution of the quantitative variables was assessed with the Shapiro–Wilk test and analyzed by T-test or Mann–Whitney test. Crude and adjusted (by age, gender, and smoking habit) receiver operating characteristic (ROC) curves were performed to evaluate (a) and (t) MMP-8 diagnostic accuracy. The cut-off points were determined by Youden′s Index. Statistical significance was considered if *p* < 0.05.

## 3. Results

Thirty-one participants (13 healthy and 18 patients with periodontitis) took part in the study. Both groups were similar in gender and smoking status (*p* > 0.05), while age was significantly higher in participants affected by periodontitis, in comparison with the healthy volunteers (54.1 ± 8.5 and 43.7 ± 14.0 years, respectively; *p* = 0.02). Regarding clinical parameters, CAL (means ± SD) was 1.6 ± 0.4 mm and 6.3 ± 2.2 mm in control and periodontitis groups, respectively (*p* < 0.0001), while PPD measurements were 2.2 ± 0.3 and 5.4 ± 1.6 mm in the same groups (*p* < 0.0001) (Table 1). None of the healthy sites and 78.2% of periodontitis sites showed a positive BOP (data not shown).

**Figure 1:**
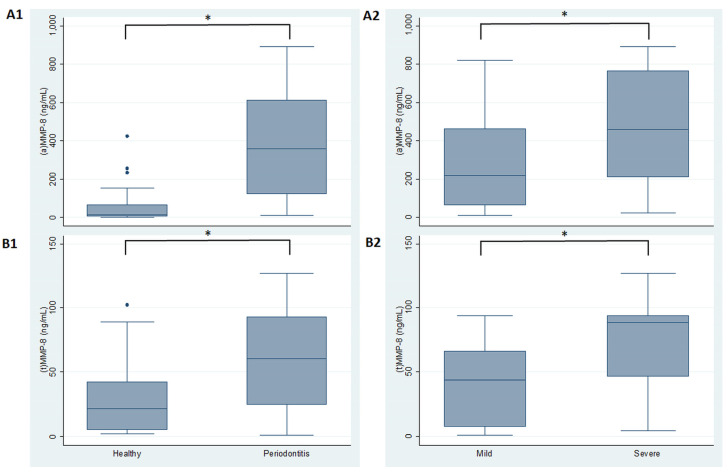
MMP-8 levels in healthy and periodontitis sites. (**A1**) Active and (**A2**) total MMP-8 levels by diagnosis. (**B1**) Active and (**B2**) total MMP-8 levels by disease periodontitis severity. (a) active; (t) total. * *p* < 0.0001.

In the analysis of MMP-8 tests according to periodontal site diagnosis and severity, (a) MMP showed a higher level (median (interquartile range )) in periodontitis sites (357.6 (490.22 ng/mL)) in comparison to healthy sites (13.5 (62.11 ng/mL)), with statistically significant differences (*p* < 0.001). Similarly, (t) MMP-8 showed higher levels in periodontitis versus healthy sites (60.62 (68.09 ng/mL) and 21.54 (37.20 ng/mL), respectively) (*p* < 0.001). When comparing MMP-8 forms between different sites affected by periodontitis, it was observed that (a) MMP-8 was greater in severe (458.84 (552.51 ng/mL)) than mild sites (218.57 (399.92 ng/mL)), with statistically significant differences (*p* < 0.001). Accordingly, total MMP-8 presented the highest levels in severe sites (88.369 (47.09 ng/mL)), compared to mild (43.89 (58.60 ng/mL)) sites, with statistically significant differences (*p* < 0.001) (Figure 1).

**Figure 2:**
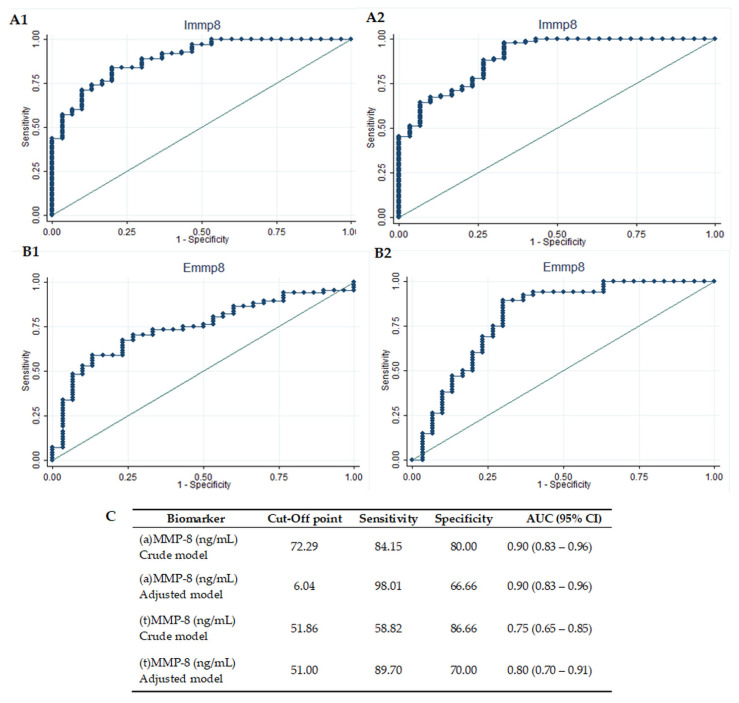
Diagnostic potential of MMP-8 tests to identify healthy sites. Diagnostic potential of active metalloproteinase-8 (a) MMP-8, to identify healthy from periodontitis sites, crude (**A1**) and adjusted (**A2**) models; diagnostic potential of (t) MMP-8 to identify healthy from periodontitis sites, crude (**B1**) and adjusted (**B2**) models; (**C**) sensitivity, specificity, and cut-off points of (a) MMP-8 and (t) MMP-8 diagnostic potential to identify healthy from periodontitis sites. AUC: area under the curve. CI: confidence interval. (a) MMP-8: active matrix metalloproteinase-8; (t) MMP-8: total matrix metalloproteinase-8. Immp8: IFMA MMP-8; Emmp8: ELISA MMP-8.

**Figure 3:**
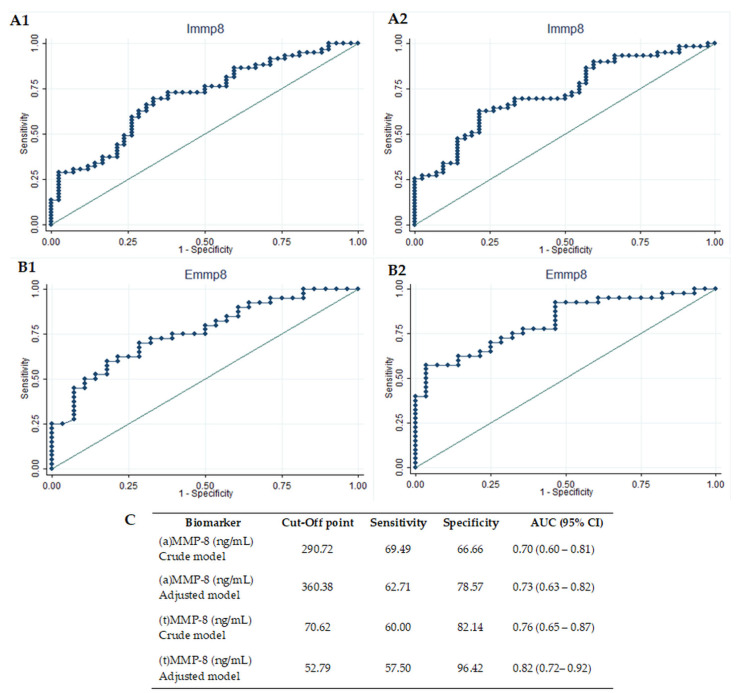
Diagnostic potential of biomarkers to identify severe periodontitis sites. Diagnostic potential of (a) MMP-8 to identify mild from severe periodontitis sites, crude (**A1**) and adjusted (**A2**) models; diagnostic potential of (t) MMP-8 to identify mild from severe periodontitis sites, crude (**B1**) and adjusted (**B2**) models; (**C**) sensitivity, specificity and cut-off points of (a) MMP-8 and (t) MMP-8. AUC: area under the curve. CI: confidence interval. (a) MMP-8: active matrix metalloproteinase-8; (t) MMP-8: total matrix metalloproteinase-8. Immp8: IFMA MMP-8; Emmp8: ELISA MMP-8.

Figure 2 and Figure 3 illustrate the diagnostic accuracy of ELISA (total MMP-8) and IFMA (active MMP-8) tests for periodontitis diagnosis and severity at the site level. ROC curves presented a high diagnostic accuracy for the discrimination of healthy from periodontitis sites in the (a) MMP-8 crude (AUC = 0.90, 95% CI 0.83–0.96) and adjusted models (AUC = 0.90, 95% CI 0.83–0.96). Total MMP-8 showed a high diagnostic precision in the adjusted model (AUC = 0.80, 95% CI 0.70–0.91), while a lower, but significant, performance was accomplished by the (t) MMP-8 crude model (AUC = 0.75, 95% CI 0.65–0.85). Optimal cut-off points to discriminate healthy sites from diseased sites were calculated with Youden′s index. The active MMP-8-adjusted model achieved the best performance, with a sensitivity of 98% and a specificity of 67%, at a cut-off point of 6.04 ng/mL. The total MMP-8-adjusted model, on the other hand, presented a sensitivity of 90% and a specificity of 70%, at a cut-off point of 51 ng/mL (Figure 2).

Concerning the identification of mild to severe periodontitis sites (Figure 3), the (t) MMP-8-adjusted model demonstrated a high diagnostic precision, defined by ROC curves with AUC ≥ 0.80 (AUC = 0.81, 95% CI 0.72–0.92). The active MMP-8 crude model (AUC = 0.703, 95% CI 0.601–0.805), (a) MMP-8-adjusted model (AUC = 0.73, 95% CI 0.63–0.82) and (t) MMP-8 crude model (AUC = 0.76, 95% CI 0.65–0.87), on the other hand, reached a regular performance. When optimal cut-off points were calculated by Youden′s Index, the (t) MMP-8-adjusted model achieved the best performance, with a sensitivity of 58% and a specificity of 96%, at a cut-off point of 52.79 ng/mL. In the second place, the (t) MMP-8 crude model achieved a sensitivity of 60% and a specificity of 82% (at a cut-off point of 70.62 ng/mL), while the (a) MMP8-adjusted model was 63% sensitive and a 79% specific in discriminating periodontitis severity, with a cut-off point of 360.38 ng/mL (Figure 3).

Finally, the correlation between total and active MMP-8 in all samples was disaggregated according to the diagnosis and periodontitis severity of the sites (H versus Ps and M versus S). A positive correlation was found between (a) MMP-8 and (t) MMP-8, obtained by ELISA and IFMA, respectively, in the total samples and each subgroup (*p* < 0.001) (Table 2).

## 4. Discussion

Periodontitis is a bacterially triggered, immunoinflammatory disease. Even when clinical parameters are the gold standard for periodontal disease screening, promissory objective biomarkers studied in oral fluids might aid in periodontal disease diagnosis, severity, classification, and monitoring [2,3]. MMP-8, the main gingival collagenase, is related to the periodontal status, severity, and progression, representing the most studied biomarker for the diagnosis of periodontitis in gingival crevicular fluid [11,12,24]. However, different MMP-8 measurement methods with different levels of agreement may limit its applicability as an adjunctive tool for periodontal disease screening [25]. Herein, we show that (a) MMP-8 had the highest accuracy to discriminate between healthy and periodontitis sites, whilst (t) MMP-8 demonstrated the best diagnostic precision in the detection of mild from severe periodontitis sites (AUC ≥ 0.80). As expected, we found significantly higher levels of MMP-8 in periodontitis versus healthy subjects and severe than mild sites, measured both by IFMA and ELISA methods. This can be explained, at least in part, due to the fact that the more MMP-8 is converted to its active form, the more clinically active, progressive or up-graded periodontitis is [6,13,16,26,27,28,29,30,31].

Both assays were also able to discriminate according to periodontal diagnosis and severity in crude and adjusted models by gender, sex, and age.

Even when total and active MMP-8 levels were raised in periodontitis, compared to gingivitis and healthy sites in the literature, active MMP-8, detected by IFMA, was a better predictor of periodontal status in previous works [16,25,32]. In fact, the active MMP-8 type was predominant in periodontitis, whereas the latent forms were mainly associated with gingivitis in some studies [16,17,25,26,27]. IFMA was more effective in the detection of periodontitis, whereas MMP-8 recorded by ELISA was scarcely detected in GCF from healthy sites [25,32]. IFMA also reported a 63.1% of MMP-8 reduction after periodontal therapy of periodontitis sites, while MMP-8 post-therapy changes recorded by ELISA were not significantly different [16].

The aforementioned assays use different antibodies for MMP-8 detection, and the identification of active (free and complex) and latent (zymogenic) forms of MMP-8 might not always correlate with periodontal status and severity. The active form of MMP-8 is the main factor responsible for periodontal connective tissue destruction, while total MMP-8 has shown controversial outcomes. In general, active MMP-8 levels seem to be more accurate than total MMP-8 measurements in the screening of periodontal disease [6,26,28,29], and it could be recommended in oral fluids as a diagnostic biomarker for periodontal disease [33,34,35].

Unlike former results [16,32,36], we found that MMP-8 levels detected both by ELISA and IFMA were correlated, even when disaggregating the groups between periodontitis and healthy sites. Indeed, both conventional methods constitute the gold standard for MMP-8 laboratory detection; however, they are costly and time-consuming. New faster, qualitative, and quantitative non-invasive point-of-care technologies are being studied in oral fluids and serum [37].

IFMA has shown a good agreement with MMP-8 point-of-care tests that use the same monoclonal antibody, such as dentoELISA and the matrix metalloproteinase specific chair-side dipstick, developed by Sorsa et al. [16,18,33,38]. The metalloproteinase-8 immunochromatographic chair-side dip-stick test was highly sensitive and specific in differentiating healthy and gingivitis sites from periodontitis sites [33]. Moreover, recent publications addressed the performance of the aMMP-8 point-of-care mouth rinse test, reporting its utility as an adjunctive method to classify periodontal disease according to the new classification system and to improve its opportune identification in undiagnosed patients [13,30,31].

The use of active and total MMP-8 could represent a useful adjunctive tool for the diagnosis and severity of periodontal disease. These results substantiate the applicability of new point-of-care methods, whose contribution to prevention, screening, and monitoring must be investigated. They may represent a useful aid within the framework of the new periodontal disease classification system, or even in the monitoring and referral of other inflammatory diseases, linked both to periodontitis and MMP-8, such as peri-implantitis, diabetes, and cardiovascular diseases [39,40].

## Figures and Tables

**Table 1:** Demographic characteristics of study participants.

Parameter	Healthy (*n* = 13)	Periodontitis (*n* = 18)	*p*
Age	43.7 ± 14.0	54.1 ± 8.5	0.02
Gender (Females)	7	12	>0.05
Smoking	2	3	>0.05
CAL (mm)	1.6 ± 0.4	6.3 ± 2.2	<0.0001
PPD (mm)	2.2 ± 0.3	5.4 ± 1.6	<0.0001

Data presented as mean ± standard deviation or absolute frequencies. CAL: clinical attachment level; PPD: periodontal probing depth; BOP: bleeding on probing.

**Table 2:** Correlation between (a) and (t) MMP-8 disaggregated by diagnosis and severity.

Biomarker	All Samples (a) MMP-8	H (a) MMP-8	Ps (a) MMP-8	M (a) MMP-8	S (a) MMP-8
(t) MMP-8 (r)	0.85	0.92	0.80	0.83	0.66

Results expressed as *p*-values; bold: *p* < 0.001 and estimate. H: healthy sites; Ps: periodontitis sites; M: mild periodontitis sites; S: severe periodontitis sites; (a) MMP-8: active matrix metalloproteinase-8; (t) MMP-8: total matrix metalloproteinase-8. Spearman′s correlation test, *p* < 0.0001.

## References

[1] Baehni P., Tonetti M.S. (2010). On behalf of Group 1 of the European Workshop on Periodontology Conclusions and consensus statements on periodontal health, policy and education in Europe: A call for action-consensus view 1. Eur. J. Dent. Educ..

[2] Tonetti M.S., Greenwell H., Kornman K.S. (2018). Staging and grading of periodontitis: Framework and proposal of a new classification and case definition. J. Periodontol..

[3] Buduneli N., Kinane D.F. (2011). Host-derived diagnostic markers related to soft tissue destruction and bone degradation in periodontitis. J. Clin. Periodontol..

[4] Giannobile W. (1997). Crevicular fluid biomarkers of oral bone loss. Curr. Opin. Periodontol..

[5] Armitage G.C. (2004). Analysis of gingival crevice fluid and risk of progression of periodontitis. Periodontol. 2000.

[6] Sorsa T., Gursoy U.K., Nwhator S., Hernández M., Tervahartiala T., Leppilahti J., Gürsoy M., Könönen E., Emingil G., Pussinen P. (2015). Analysis of matrix metalloproteinases, especially MMP-8, in gingival crevicular fluid, mouthrinse and saliva for monitoring periodontal diseases. Periodontol. 2000.

[7] Franco C., Patricia H.-R., Timo S., Claudia B., Marcela H. (2017). Matrix Metalloproteinases as Regulators of Periodontal Inflammation. Int. J. Mol. Sci..

[8] Golub L.M., Lee H.M., Greenwald R.A., Ryan M.E., Sorsa T., Salo T., Giannobile W. (1997). A matrix metalloproteinase inhibitor reduces bone-type collagen degradation fragments and specific collagenases in gingival crevicular fluid during adult periodontitis. Inflamm. Res..

[9] Ingman T., Tervahartiala T., Ding Y., Tschesche H., Haerian A., Kinane D.F., Konttinen Y.T., Sorsa T. (1996). Matrix metalloproteinases and their inhibitors in gingival crevicular fluid and saliva of periodontitis patients. J. Clin. Periodontol..

[10] Ingman T., Sorsa T., Suomalainen K., Halinen S., Lindy O., Lauhio A., Saari H., Konttinen Y.T., Golub L.M. (1993). Tetracycline Inhibition and the Cellular Source of Collagenase in Gingival Crevicular Fluid in Different Periodontal Diseases. A Review Article. J. Periodontol..

[11] Hernández M., Baeza M., Contreras J., Sorsa T., Tervahartiala T., Valdés M., Chaparro A., Hernández-Ríos P. (2020). MMP-8, TRAP-5, and OPG Levels in GCF Diagnostic Potential to Discriminate between Healthy Patients’, Mild and Severe Periodontitis Sites. Biomolecules.

[12] Baeza M., Garrido M., Hernández-Ríos P., Dezerega A., García-Sesnich J., Strauss F., Aitken J.P., Lesaffre E., Vanbelle S., Gamonal J. (2016). Diagnostic accuracy for apical and chronic periodontitis biomarkers in gingival crevicular fluid: An exploratory study. J. Clin. Periodontol..

[13] Sorsa T., Alassiri S., Grigoriadis A., Räisänen I.T., Pärnänen P., Nwhator S.O., Gieselmann D.-R., Sakellari D. (2020). Active MMP-8 (aMMP-8) as a Grading and Staging Biomarker in the Periodontitis Classification. Diagnostics.

[14] Arias-Bujanda N., Regueira-Iglesias A., Balsa-Castro C., Nibali L., Donos N., Tomás I. (2019). Accuracy of single molecular biomarkers in gingival crevicular fluid for the diagnosis of periodontitis: A systematic review and meta-analysis. J. Clin. Periodontol..

[15] Sorsa T., Tjäderhane L., Konttinen Y.T., Lauhio A., Salo T., Lee H., Golub L.M., Brown D.L., Mäntylä P. (2006). Matrix metalloproteinases: Contribution to pathogenesis, diagnosis and treatment of periodontal inflammation. Ann. Med..

[16] Sorsa T., Hernández M., Leppilahti J., Munjal S., Netuschil L., Mäntylä P. (2009). Detection of gingival crevicular fluid MMP-8 levels with different laboratory and chair-side methods. Oral Dis..

[17] Teles R., Sakellari D., Teles F., Konstantinidis A., Kent R., Socransky S., Haffajee A. (2010). Relationships Among Gingival Crevicular Fluid Biomarkers, Clinical Parameters of Periodontal Disease, and the Subgingival Microbiota. J. Periodontol..

[18] Hanemaaijer R., Sorsa T., Konttinen Y.T., Ding Y., Sutinen M., Visser H., van Hinsbergh V.W.M., Helaakoski T., Kainulainen T., Rönkä H. (1997). Matrix Metalloproteinase-8 Is Expressed in Rheumatoid Synovial Fibroblasts and Endothelial Cells. J. Biol. Chem..

[19] Steen P.E.V.D., Wuyts A., Husson S.J., Proost P., Van Damme J., Opdenakker G. (2003). Gelatinase B/MMP-9 and neutrophil collagenase/MMP-8 process the chemokines human GCP-2/CXCL6, ENA-78/CXCL5 and mouse GCP-2/LIX and modulate their physiological activities. JBIC J. Biol. Inorg. Chem..

[20] Hernández M., Martínez B., Tejerina J.M., Valenzuela M.A., Gamonal J. (2007). MMP-13 and TIMP-1 determinations in progressive chronic periodontitis. J. Clin. Periodontol..

[21] Chapple I.L., Mealey B.L., Van Dyke T.E., Bartold P.M., Dommisch H., Eickholz P., Geisinger M.L., Genco R.J., Glogauer M., Goldstein M. (2018). Periodontal health and gingival diseases and conditions on an intact and a reduced periodontium: Consensus report of workgroup 1 of the 2017 World Workshop on the Classification of Periodontal and Peri-Implant Diseases and Conditions. J. Periodontol..

[22] Ríos M.H., Sorsa T., Obregón F., Tervahartiala T., Valenzuela M.A., Pozo P., Dutzan N., Lesaffre E., Molas M., Gamonal J. (2009). Proteolytic roles of matrix metalloproteinase (MMP)-13 during progression of chronic periodontitis: Initial evidence for MMP-13/MMP-9 activation cascade. J. Clin. Periodontol..

[23] Hemmilä I., Dakubu S., Mukkala V.-M., Siitari H., Lövgren T. (1984). Europium as a label in time-resolved immunofluorometric assays. Anal. Biochem..

[24] Arias-Bujanda N., Regueira-Iglesias A., Blanco-Pintos T., Alonso-Sampedro M., Relvas M., González-Peteiro M.M., Balsa-Castro C., Tomás I., Sampedro-Alonso M. (2020). Diagnostic accuracy of IL1β in saliva: The development of predictive models for estimating the probability of the occurrence of periodontitis in non-smokers and smokers. J. Clin. Periodontol..

[25] Gursoy U.K., Könönen E., Pradhan-Palikhe P., Tervahartiala T., Pussinen P., Suominen-Taipale L., Sorsa T. (2010). Salivary MMP-8, TIMP-1, and ICTP as markers of advanced periodontitis. J. Clin. Periodontol..

[26] Romanelli R., Mancini S., Laschinger C., Overall C.M., Sodek J., McCulloch C.A.G. (1999). Activation of Neutrophil Collagenase in Periodontitis. Infect. Immun..

[27] Kiili M., Cox S.W., Chen H.W., Wahlgren J., Maisi P., Eley B.M., Salo T., Sorsa T. (2002). Collagenase-2 (MMP-8) and collagenase-3 (MMP-13) in adult periodontitis: Molecular forms and levels in gingival crevicular fluid and immunolocalisation in gingival tissue. J. Clin. Periodontol..

[28] Lee W., Aitken S., Sodek J., McCulloch C.A.G. (1995). Evidence of a direct relationship between neutrophil collagenase activity and periodontal tissue destruction in vivo: Role of active enzyme in human periodontitis. J. Periodontal Res..

[29] Mancini S., Romanelli R., Laschinger C.A., Overall C.M., Sodek J., McCulloch C.A. (1999). Assessment of a Novel Screening Test for Neutrophil Collagenase Activity in the Diagnosis of Periodontal Diseases. J. Periodontol..

[30] Räisänen I.T., Lähteenmäki H., Gupta S., Grigoriadis A., Sahni V., Suojanen J., Seppänen H., Tervahartiala T., Sakellari D., Sorsa T. (2021). An aMMP-8 Point-of-Care and Questionnaire Based Real-Time Diagnostic Toolkit for Medical Practitioners. Diagnostics.

[31] Deng K., Pelekos G., Jin L., Tonetti M.S. (2021). Diagnostic accuracy of a point-of-care aMMP-8 test in the discrimination of periodontal health and disease. J. Clin. Periodontol..

[32] Leppilahti J.M., Hernández-Ríos P.A., Gamonal J.A., Tervahartiala T., Brignardello-Petersen R., Mäntylä P., Sorsa T., Hernández M. (2013). Matrix metalloproteinases and myeloperoxidase in gingival crevicular fluid provide site-specific diagnostic value for chronic periodontitis. J. Clin. Periodontol..

[33] Mäntylä P., Stenman M., Kinane D.F., Tikanoja S., Luoto H., Salo T., Sorsa T. (2003). Gingival crevicular fluid collagenase-2 (MMP-8) test stick for chair-side monitoring of periodontitis. J. Periodontal Res..

[34] Ramseier C.A., Kinney J.S., Herr A., Braun T., Sugai J.V., Shelburne C.A., Rayburn L.A., Tran H.M., Singh A.K., Giannobile W.V. (2009). Identification of Pathogen and Host-Response Markers Correlated With Periodontal Disease. J. Periodontol..

[35] Salminen A., Gursoy U.K., Paju S., Hyvärinen K., Mäntylä P., Buhlin K., Könönen E., Nieminen M.S., Sorsa T., Sinisalo J. (2014). Salivary biomarkers of bacterial burden, inflammatory response, and tissue destruction in periodontitis. J. Clin. Periodontol..

[36] Leppilahti J.M., Kallio M.A., Tervahartiala T., Sorsa T., Mäntylä P. (2014). Gingival Crevicular Fluid Matrix Metalloproteinase-8 Levels Predict Treatment Outcome Among Smokers With Chronic Periodontitis. J. Periodontol..

[37] Lorenz K., Keller T., Noack B., Freitag A., Netuschil L., Hoffmann T. (2016). Evaluation of a novel point-of-care test for active matrix metalloproteinase-8: Agreement between qualitative and quantitative measurements and relation to periodontal inflammation. J. Periodontal Res..

[38] Sorsa T., Mäntylä P., Ronka H., Kallio P., Kallis G.-B., Lundqvist C., Kinane D.F., Salo T., Golub L.M., Teronen O. (1999). Scientific Basis of a Matrix Metalloproteinase-8 Specific Chair-side Test for Monitoring Periodontal and Peri-implant Health and Disease. Ann. N. Y. Acad. Sci..

[39] Al-Majid A., Alassiri S., Rathnayake N., Tervahartiala T., Gieselmann D.-R., Sorsa T. (2018). Matrix Metalloproteinase-8 as an Inflammatory and Prevention Biomarker in Periodontal and Peri-Implant Diseases. Int. J. Dent..

[40] Dejonckheere E., Vandenbroucke R., Libert C. (2011). Matrix metalloproteinase8 has a central role in inflammatory disorders and cancer progression. Cytokine Growth Factor Rev..

